# Influence of Intensity on Post-Running Jump Potentiation in Recreational Runners vs. Physically Active Individuals

**DOI:** 10.5114/jhk/172268

**Published:** 2023-10-11

**Authors:** Cristiano Rafael Moré, Rita Adriana Stoeterau Moré, Daniel Boullosa, Rodolfo André Dellagrana

**Affiliations:** 1 Graduate Program in Movement Sciences, Federal University of Mato Grosso do Sul, Campo Grande, MS, Brazil.; 2 Faculty of Physical Activity and Sports Sciences, Universidad de León, León, Spain.; 3 College of Healthcare Sciences, James Cook University, Townsville, Australia.; 4 Physical Education Department, State University of Ponta Grossa, Ponta Grossa, PR, Brazil.

**Keywords:** running, performance, conditioning activity, jump, sprint

## Abstract

The aim of this study was to verify post-activation performance enhancement (PAPE) in jumping and sprinting after two endurance volume-equated running protocols with different intensities, in runners vs. active individuals. Nine recreational runners (age: 34.5 ± 9.3 years, body mass: 73.1 ± 11.9 kg, body height: 1.76 ± 0.06 m, 17.4 ± 4.4 %body fat; maximum aerobic speed [MAS]: 16.4 ± 1.0 km•h^−1^), and 9 active individuals (age: 34.1 ± 9.4 years; body mass: 83.2 ± 7.7 kg; body height: 1.79 ± 0.06 m; 25.6 ± 5.4 %body fat; MAS: 13.3 ± 1.2 km•h^−1^) volunteered for participation. The evaluations were performed over three days as follows: 1) anthropometric measures, physical fitness tests, and the University of Montreal Track Test (UMTT) to determine MAS and the distance to be covered in the running protocols; 2 and 3) the countermovement jump (CMJ) and the flying 20-m sprint (SPRINT) were assessed pre- and post-running at 70% of MAS or a time trial race (TTR), equated by volume and completed in random order. A three-way ANOVA (time*group*running) was performed to analyze the PAPE effects. The results showed a time effect (F = 10 .716; p < 0.01) and a group*running interaction (F = 12.094; p < 0.01) for the CMJ, indicating that active individuals demonstrated PAPE after running at 70% of MAS, while for runners both running interventions (70% of MAS and TTR) induced PAPE in CMJ performances. For the SPRINT, a time*group interaction (F = 4.790; p = 0.044) and a group effect were observed, with runners showing greater SPRINT performances than active individuals. From the current results, it can be suggested that training background and intensity can modulate PAPE responses in jumping and sprinting after volume-equated running protocols at different intensities.

## Introduction

Post-activation performance enhancement (PAPE) is defined as a transient and acute improvement in the performance of a physical task, just after a conditioning activity (CA) (Cuenca-Fernandéz et al., 2017). PAPE has been traditionally applied in power exercises (Boullosa et al., 2018; Chameraet al., 2023; Kobal et al., 2019), since CA with maximal or near maximal intensities may increase power production in the subsequent exercise (Seitz and Haff, 2016). On the other hand, several studies have observed an enhancement in power performance after different running exercises in endurance athletes (Boullosa and Tuimil, 2009; Chroboczek et al., 2021; García-Pinillos et al., 2015, 2018; Gervasi et al., 2018). Therefore, practitioners in endurance sports can also benefit from PAPE, since research has been shown that prolonged activities could promote a greater jump performance, strength and power outputs (Boullosa et al., 2018). The most used tests to verify PAPE effects in endurance athletes are the countermovement jump (CMJ) and linear sprints (e.g., 10 to 30 m) (Boullosa et al., 2011; Del Rosso et al., 2016; García-Pinillos et al., 2015; Spieszny et al., 2022).

Mechanisms behind PAPE have been suggested to be muscle temperature changes, changes in muscle water content, increased excitability of motoneurons, increased recruitment of motor units, acute elevations in plasma catecholamines levels, increases in testosterone concentration, learning and familiarization effect, as well the phosphorylation of the myosin regulatory light chains (RLC) related to post-activation potentiation (PAP) (Blazevich and Babault, 2019; Zimmermann et al., 2020). For endurance athletes, better balance between fatigue and potentiation could be expected, since slow-twitch fibers present greater fatigue resistance (Boullosa et al., 2018), in which Ca^2+^ sensitivity seems to be maximized at low Ca^2+^ levels and limited at saturated Ca^2+^ levels (Sale, 2002). Therefore, it has been suggested that prolonged activities (i.e., > 1 min) would induce a greater power performance as consequence of a better potentiation and fatigue relationship (Boullosa et al., 2018).

Noteworthy, the magnitude of PAPE depends on the balance between fatigue and potentiation, which is related to some factors including individual physical fitness (Chiu and Barnes, 2003; Guerra Junior et al., 2020), athlete’s training status (Wilson et al., 2013), the endurance running performance level (Del Rosso et al., 2021), the rest period after CA (Wilson et al., 2013), and intensity of CAs (Seitz and Haff, 2016; Wilson et al., 2013). An enhancement in performance occurs when potentiation overlaps fatigue, however, performance decreases when fatigue overlaps the potentiation (Rassier and Macintosh, 2000). In this context, according to Sale (2002), it can be postulated that the conditioning level, volume and intensity of the CA are the most important factors that influence the magnitude of balance between fatigue and potentiation and subsequent PAPE.

Understanding the best CA protocols and loading factors to PAPE in endurance runners is still unclear. While some previous studies have reported improvements in CMJ performances after maximal incremental running protocols (Boullosa and Tuimil, 2009; Boullosa et al., 2011; García-Pinillos et al., 2018), others have observed CMJ improvements after submaximal running at 80% of velocity associated to VO_2MAX_ (Vuorimaa et al., 2006), 85–100% of maximum aerobic speed (MAS) (García-Pinillos et al., 2015) or during a 30 km self- paced running trial (Del Rosso et al., 2016). Importantly, only one study (Boullosa and Tuimil, 2009) has reported enhancement in CMJ performances after two maximal running protocols (incremental vs. time limit at MAS), which were not observed in non-runners, thus suggesting that these PAPE responses after endurance running exercises are specific for endurance runners.

Neuromuscular factors have shown a significant influence on endurance performance since both strength and power training seem to improve running economy (Millet et al., 2002; Saunders et al., 2006) and, consequently, endurance performance (Paavolainen et al., 1999). The evaluation of power after running activities can be helpful for coaches aiming to monitor the training status of their athletes. Thus, the aim of this study was to verify the effects of two running protocols differing in intensity on PAPE responses of individuals with different training background (i.e., runners vs. non-runners). Based on previous literature, we hypothesized that higher running intensities would induce greater PAPE responses in runners when compared to non-runners because of their greater resistance to fatigue.

## Methods

### Participants

**Figure 1:**
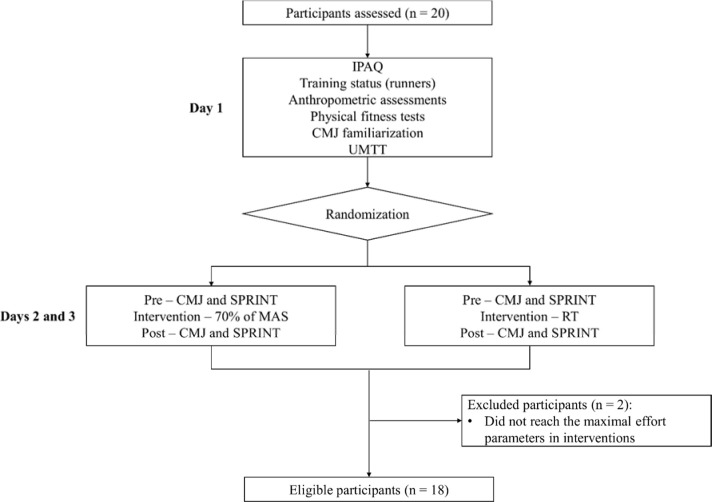
Experimental study design.

Twenty male participants volunteered for this study and were divided into two groups: 10 recreational runners and 10 physically active individuals. The training status of recreational runners was as follows: a) experience in endurance training (10.3 ± 7.8 years); b) training weekly volume of 55.5 ± 46.7 km; c) training weekly frequency of 4.1 ± 1.16 days; and d) best time in official 5 km races of 20.6 ± 1.9 min. The inclusion criteria adopted for the recreational runners were: a) familiarized with medium and long-distance running; b) training routine with 3–5 running sessions per week in the last six months; c) running pace < 5 min/km; d) frequent participation in medium and long running competitions (at least two races in the last 6 months). The inclusion criterion for physically active individuals was solely being “active”, according to the physical activity level classification (IPAQ). The following exclusion criteria were established for both groups: a) lower limb injuries in the last two months; b) taking ergogenic supplements; c) participation in other research; and d) not achieving the parameters of maximum effort in the running tests (≤ 90% of the maximum heart rate (HR_MAX_) in the University Montreal Track Test (UMTT), ≤ 85% of the HR_MAX_ in the time trial race (TTR) intervention, and rating of perception exertion (RPE) ≥ 18). Therefore, 18 participants divided into 9 runners (34.5 ± 9.3 years; 73.1 ± 11.9 kg; 1.76 ± 0.06 m; 17.4 ± 4.4 %body fat; and 16.4 ± 1.0 km·h^−1^/MAS) and 9 active individuals (34.1 ± 9.4 years; 83.2 ± 7.7 kg; 1.79 ± 0.06 m; 25.6 ± 5.4 %bodyfat; 13.3 ± 1.2 km·h^−1^/MAS) were eligible to take part in this study (Figure 1). All participants provided written informed consent. Ethical approval (28 April 2021) was obtained from the Human Research Committee of the Federal University of Mato Grosso do Sul (CAAE: 44522821.4.0000.0021).

### Design and Procedures

The study protocol was performed on three different days at the same hour within a maximum of two weeks between sessions (Figure 1). On the day before each test, participants were suggested to avoid vigorous exercises of the lower limbs, and alcohol and caffeine intake. On the first day, several assessments were performed: a questionnaire related to training status (only for runners), the Portuguese version of the International Physical Activity Questionnaire (IPAQ) (Matsudo et al., 2012), anthropometric assessment, physical fitness tests (a handgrip test, a sit-and-reach flexibility test, a 20-m sprint, University Montreal Track Test [UMTT] incremental running), and the familiarization with the CMJ. The CMJ familiarization consisted of three sets of ten submaximal CMJ on the ground with a 1-min rest interval in between. The UMTT was used to measure maximum aerobic speed (MAS) and the distance to be covered in the next endurance running interventions (70% of MAS and TTR). The second and third sessions consisted of two protocols equated by the total distance covered in the UMTT. Thus, participants performed a warm-up (moderate intensity running, with 60% of HR_MAX_), during 10 min. Afterwards, participants performed two CMJs, separated by 15 s, and after 1 min, they performed two 20-m sprints (SPRINT), separated by 1-min rest periods in order to determine baseline levels (pre-intervention). As soon as possible, after baseline tests, participants completed intervention protocol: the distance covered in the UMTT at 70% of MAS or a time trial race (TTR), which were randomized using the “random” function in Excel software. After 2 min of recovery, participants underwent post-intervention assessment (the same assessments as at baseline: two CMJs and two SPRINTs). The environmental factors were monitored using data from the Local State Weather and Climate Monitoring Center, in which the CV between days was less than 15% for temperature (29.2 ± 3.2 °C).

### Measures

#### Physical Activity Assessment

The International Physical Activity Questionnaire (IPAQ) (Matsudo et al., 2012) was used to measure the participants' physical activity level. The questionnaire was fulfilled by participants who received verbal guidance. Participants were considered “physically active” when meeting one of the following criteria: a) vigorous physical activity: ≥ 3 days/week and ≥ 20 minutes/session; b) moderate physical activity or walking ≥ 5 days/week and ≥ 30 minutes/session; c) any activity combined ≥ 5 days/week and ≥ 150 min/week.

#### Training Status Assessment

A questionnaire was applied only to recreational runners, with questions including quantitative data related to training (i.e., frequency, intensity and volume of training), as well as competitive experience in medium and long-distance running events.

#### Anthropometric Assessment

Body mass was measured on a scale with 0.1 kg accuracy (Soehnle®, Murrhardt, Germany). Body height was measured with a stadiometer with 0.1 cm accuracy (Sanny®, Standard model, São Paulo, Brazil). The body mass index (BMI) was calculated using the equation: BMI = body mass/height^2^ (kg/m^2^). The skinfolds thickness was measured at seven body sites (chest, mid-axillary, triceps, subscapular, abdominal, suprailiac, and thigh), using a calibrated caliper (Cescorf®, São Paulo, Brazil), with 0.1 mm accuracy. Body density was calculated using the equation described by Jackson and Pollock (1978), and the body fat percentage was calculated using the Siri equation (Siri, 1961).

#### Physical Fitness Assessments

Handgrip strength was measured with a dynamometer (Saehan Corporation, 973, Yangdeok-Dong, Masan 630-728, Korea) adjusted according to the size of the participants’ hand. The test was performed with participants seated, elbow flexed at 90° and forearm placed in a neutral position. Three attempts were made for each participant, and the highest value was used for further analyses.

Flexibility was measured with the sit-and-reach test. The test was administered using a box with a height of 30.5 cm and a depth of 56.5 cm. A reach distance of 23 cm corresponded with the position of the feet against the box. The test was performed according to the classic protocol described by Wells and Dillon (1952).

Participants undertook a 20-m sprint test (SPRINT) on an outdoor 400-m track. The time was recorded by photocells (Elite Speed®, São Paulo, Brazil) at the start lines and the finish lines. Each participant completed two trials separated by 1 min of rest. Participants decided themselves when to start the test from a static position 20 m behind the photocell. The best time recorded over the 20-m distance from a flying start was used for further analyses.

The University of Montreal Track Test (UMTT) was used to measure aerobic fitness through maximal aerobic speed (MAS). The UMTT was performed in accordance with the original protocol developed by Léger and Boucher (1980) on an official outdoor 400-m track. The initial speed was set at 8 km•h^−1^, which was subsequently increased by 1 km•h^−1^ every 2 min until voluntary exhaustion. The speed of each stage was controlled by an experienced cyclist who dictated the pace using a speedometer calibrated according to the manufacturer's recommendations (Edge 520; Garmin, Taiwan). The speed of the final stage completed was considered to determine MAS. If a stage could not be completed to its full length in the test, MAS was calculated according to the Kuipers’ equation (Kuipers et al., 2003).

#### Pre- and Post-Intervention Assessments

To verify post-activation performance enhancement (PAPE), pre- and post-interventions, CMJ and SPRINT tests were performed. First, participants performed two CMJs separated by 15 s, with the highest jump height used as a baseline condition (pre-intervention). Jump height (JH) was calculated with the flight time method, using a contact mat (Jump System Pro, Cefise, São Paulo, Brazil). Subsequently, participants performed two SPRINT tests (see description of the test in the next sections) with at least 1-min rest intervals between attempts. The test procedures were the same as previously described with the best time of the SPRINT considered for further analysis (Boullosa et al., 2011). In the post intervention, following 2 min of recovery, participants performed two CMJs and two SPRINTs, according to the procedures previously described.

#### Intervention Protocols

The interventions were equated by the total distance covered in the UMTT (i.e., physically active individuals and recreational runners covered the distance reached in the UMTT [2,261 ± 572 m and 3,852 ± 558 m, respectively]). Submaximal running consisted of a constant intensity run that corresponded to 70% of MAS measured in the UMTT. The pace (70% of MAS) was imposed by a cyclist with a velocimeter calibrated in accordance with the manufacturer’s instructions (Edge 520; Garmin, Taiwan).

For the TTR intervention, participants ran the total distance covered in the UMTT, in the fastest possible time, using the pacing strategies that they considered most appropriate. All conditions were identical to the UMTT testing day including the use of the same running clothes and shoes.

During all interventions, the heart rate (HR) was monitored during the warm-up, sprint tests, and running tests (70% of MAS and TTR) with a HR monitor (Polar H9, Polar Electro Oy, Finland). The rating of perceived exertion (RPE) was measured using the BORG scale, which consists of 15 anchors scored from 6 to 20 (from extremely easy to extremely hard) (Borg, 1982).

### Statistical Analysis

Values are presented as mean and standard deviation (SD). Data normality was verified using the Shapiro-Wilk test. The Student’s *t*-test was used to compare anthropometric, physical fitness and running (distance and time) variables between groups (runners vs. physically active individuals). To assess the reliability of CMJ and SPRINT attempts between subjects the intraclass correlation coefficient (ICC) and coefficient of variation (CV) were used. The delta percentage (∆%) was applied to verify individual performance of participants by the equation: ∆% = ((Post-Pre/Pre)*100). Three-way analysis of variance of the mixed model (time factor [pre and post] * group factor [runners and physically active] * running factor [70% of MAS and TTR]) was used to compare CMJ and SPRINT performance in the pre- and post-interventions. Bonferroni post hoc was used for analyses. For assessment of general physical fitness, the Standard Ten (STEN) score based on body composition, handgrip strength, flexibility, 20-m sprint, and maximal aerobic speed values was used. The STEN score was calculated using the following equation: STEN = [(Participant’s score – Mean value)/SD]*2 + 5.5 (McGuigan, 2014). Thus, physical fitness variables presented a score of 1–10, in which all parameters were analyzed using the same scale (Guerra Junior et al., 2020). The general physical fitness score was the mean value of the STEN score from the five capacities assessed here (body composition, handgrip strength, flexibility, 20-m sprint, and MAS). The correlation between general physical fitness (STEN score), training status (only for runners) and PAPE responses (∆% of CMJ and SPRINT [pre and post interventions]) was analyzed with the Pearson's product moment correlation coefficient (r). All statistical analyses were performed using the IBM SPSS Statistics for Windows software, version 21.0 (IBM Corp. Armonk, NY, USA). The software G*Power version 3.1.9.2 (University of Kiel, Kiel, Germany) was used to determine statistical power. For all statistics, the level of significance was set at 5%.

## Results

Anthropometric and physical fitness characteristics are presented in Table 1. Significant differences were found between groups (physically active individuals and recreational runners) for body mass, the body mass index, the body fat percentage, aerobic power, and the SPRINT. Physically active individuals presented higher body mass and body fat percentage, and lower aerobic power and 20-m sprinting performance (*p*< 0.05) when compared to recreational runners. In addition, very good to excellent reliability values were found for the CMJ (physically active individuals: ICC = 0.979 [CI95%: 0.902–0.995] and CV = 2.4%; recreational runners: ICC = 0.975 [CI95%: 0.896–0.994] and CV = 1.4%) and the SPRINT (physically active individuals: ICC= 0.851 [CI95%: 0.404–0.966] and CV = 2.2%; recreational runners: ICC = 0.876 [CI95%: 0.388–0.973] and CV = 1.7%).

**Table 1:** Basic anthropometric, body composition and physical fitness variables

Variables	ACTIVE (N = 9)	RUNNERS (N = 9)	*p*-value
Age (years)	34.10 ± 9.40	34.50 ± 9.30	0.460
Body mass (kg)	83.23 ± 7.75	73.16 ± 11.94	0.024*
Body height (m)	1.79 ± 0.06	1.76 ± 0.06	0.220
BMI (kg•m^−2^)	26.01 ± 3.16	23.22 ± 2.39	0.025*
Body fat percentage (%)	25.61 ± 5.49	17.47 ± 4.48	0.001*
Handgrip strength (kgf)	48.11 ± 7.07	43.22 ± 5.60	0.061
Flexibility (cm)	24.72 ± 10.11	26.77 ± 7.27	0.313
20-m sprint (s)	2.91 ± 0.22	2.65 ± 0.11	0.002*
MAS (km•h^−1^)	13.35 ± 1.22	16.41 ± 0.97	<0.010*

BMI = body mass index; MAS = maximum aerobic speed; SD = standard deviation; NS = not significant; * = significant difference; ACTIVE = physically active individuals; RUNNERS = recreational runners

HR_MAX_ in the UMTT was 181 ± 13 bpm, in the TTR it was 178 ± 14 bpm (97.2% of HR_MAX_ in the UMTT), and in running at 70% of MAS it was 165±11 bpm (90.1% of HR_MAX_ in the UMTT). There was a significant difference (*p*< 0.001) between running at 70% of MAS (10.4 ± 1.3 km·h^−1^) and TTR (13.2 ± 1.8 km·h^−1^). In addition, distance covered (physically active individuals: 2,261 ± 572 m; and runners: 3,852 ± 558 m), time of running at 70% of MAS (physically active individuals: 14.3 ± 2.4 min, and runners: 20.2 ± 1.8 min) and TTR (physically active individuals: 11.4 ± 2.3 min, and runners: 15.7 ± 1.6 min) showed significant differences between groups (*p*< 0.05).

The reliability of the two attempts of the CMJ and the SPRINT pre- and post-running at 70% of MAS and TTR interventions presented high CCI values (CMJ: 0.979 to 0.994; and SPRINT: 0.931 to 0.981).

Table 2 presents the comparison between CMJ and SPRINT performances (pre- and post-interventions), running at 70% of MAS and TTR of physically active individuals and recreational runners. For the CMJ, a significant effect of time was found (F = 10.716; *p* = 0.005; observed power = 0.89), in which CMJ performance increased in both groups under the post-intervention condition (running at 70% of MAS and TTR). A significant interaction (Group*Running) was observed (F = 12.094; *p* = 0.003; observed power = 0.92), indicating that physically active individuals had higher CMJ enhancement after running at 70% of MAS (*p* = 0.006), while for runners, performance of the CMJ showed improvements in both interventions. For the SPRINT, a significant Time*Group interaction (F = 4.790; *p* = 0.044; observed power = 0.59) and a group effect (F = 7.672; *p* = 0.014; observed power = 0.73) were observed, indicating differences between groups in SPRINT performances (pre- and post-interventions), in which recreational runners had higher performances than physically active individuals.

**Table 2:** Comparative analysis of the results (mean ± SD) of the CMJ and the SPRINT between pre- and post-race intervention conditions (70% MAS and time trial race) and differences between the participants' status.

	70% MAS	TTR	70% MAS	TTR
	CMJ (cm)	CMJ (cm)	SPRINT (s)	SPRINT (s)
*Active*				
Pre	29.07 ± 6.53	28.08 ± 6.70	2.93 ± 0.27	3.04 ± 0.35
Post	31.06 ± 6.39	28.90 ± 6.12	2.90 ± 0.29	3.00 ± 0.27
*Runners*				
Pre	32.99 ± 6.55	33.64 ± 7.04	2.71 ± 0.15	2.65 ± 0.16
Post	34.47 ± 6.12	35.61 ± 6.10	2.72 ± 0.14	2.70 ± 0.13
*p value*				
Time	0.005*		0.986	
Group	0.119		0.014*	
Running	0.356		0.502	
Time*Group	0.740		0.044*	
Group*Running	0.003*		0.124	
Time*Running	0.490		0.558	
Time*Group*Running	0.106		0.473	

* = significant difference (p < 0.05); CMJ = counter movement jump; SPRINT = maximum speed run in 20 m; MAS = maximum aerobic speed; TTR = time trial race

**Figure 2:**
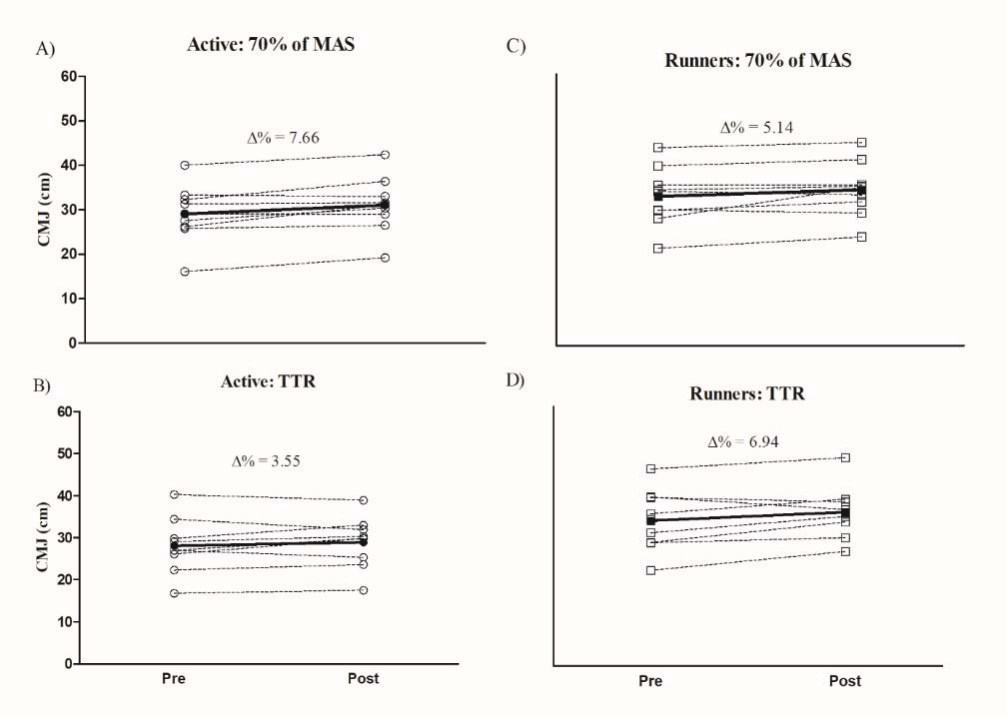
Comparison of Δ% performance in the CMJ between physically active individuals and recreational runners in: A) active: running at 70% of maximum aerobic speed (MAS); B) active: time trial race (TTR); C) runners: running at 70% of maximum aerobic speed; and D) runners: time trial race (TTR). CMJ = countermovement jump; Active = physically active individuals; Runners = recreational runners. Bold line = mean of Δ%.

**Figure 3:**
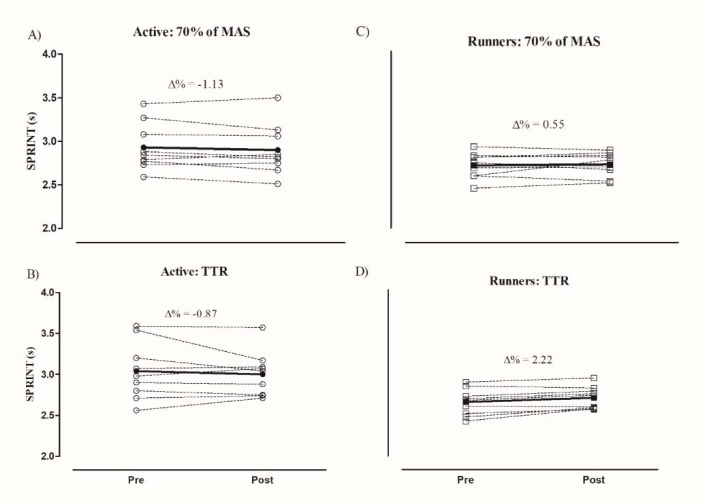
Comparison of Δ% SPRINT performance between physically active individuals and recreational runners in: A) active: running at 70% of maximum aerobic speed (MAS); B) active: time trial race (TTR); C) runners: running at 70% of maximum aerobic speed; and D) runners: time trial race (TTR). CMJ = countermovement jump; Active = physically active individuals; Runners = recreational runners. Bold line = mean of Δ%.

Values of ∆% for the CMJ and SPRINT pre- and post-interventions are presented in Figures 2 and 3. Differences between ∆% (CMJ, SPRINT) groups were not significant. For physically active participants, ∆% of the CMJ in running at 70% of MAS and TTR was 7.66 ± 7.86% and 3.55 ± 7.73% (*p*= 0.101), respectively, while for runners, ∆% of the CMJ in running at 70% of MAS and TTR was 5.14 ± 8.46% and 6.94 ± 8.69% (*p* = 0.519), respectively. For SPRINT speeds, negative values of ∆% were observed after the two interventions for physically active individuals (70% of MAS: −1.13 ± 2.38%; TTR: −0.87 ± 4.70%, *p* = 0.896), while for runners, positive values of ∆% were observed following both interventions (70% of MAS: 0.55 ± 2.99%; TTR: 2.22 ± 2.13%, *p* = 0.059).

There were no significant correlations between general physical fitness (STEN score) and PAPE performances (Table 3). However, for runners, training experience (r = 0.66, *p*< 0.05) and training volume (r = 0.67) were positively related with ∆% CMJ in the TTR.

**Table 3:** Correlation of PAPE (∆%CMJ and ∆%SPRINT) with physical fitness and training status.

	∆%CMJ in the 70% MAS run	∆%CMJ in the TTR	∆%SPRINT in the 70% MAS run	∆%SPRINT in the TTR
*Physical fitness*				
STEN score	−0.02	−0.36	0.23	0.14
*Training status*				
Training experience	0.20	0.66*	0.47	0.46
Training volume	0.19	0.67*	0.25	0.43
Training frequency	0.41	0.55	−0.25	−0.07
Training intensity	−0.05	−0.49	−0.18	−0.24

* = significant correlation (p < 0.05).

## Discussion

The aim of the present study was to verify the effects of the task (running at different intensities) and individuals’ characteristics (different conditioning levels) on PAPE responses. The first finding of this study was PAPE observed in jumping capacity, after running at 70% of MAS and TTR interventions. These results are similar to previous studies, which also used prolonged running as intervention protocols (Boullosa et al., 2011; Del Rosso et al., 2016; García-Pinillos et al., 2015, 2018). Furthermore, for runners, PAPE was observed after both interventions (running at 70% of MAS and TTR), whereas for physically active individuals, PAPE was only observed after running at 70% of MAS. Previous studies have suggested that trained individuals of different conditioning levels can benefit from PAPE after prolonged running exercises (Boullosa et al., 2018).

Significant differences were confirmed for intensity, duration and distance, when comparing both running interventions (70% of MAS and TTR). The enhancement in CMJ performances after running interventions were significantly different only for physically active individuals, where a significant CMJ improvement (∆% = 7.66, *p*< 0.05) was demonstrated for running at 70% of MAS, while no significant CMJ improvement was observed for TTR (∆% = 3.55, *p*> 0.05). These results suggest that non-runners may also benefit from PAPE after a non-fatiguing CA. Previously, Chiu and Barnes (2003) observed an impairment (1 to 4%) in jump performance (i.e., drop jump) for physically active individuals after a CA performed at 90% of the 1RM (five sets / 1 repetition), whereas for trained individuals, jump performance improved (1 to 3%). Although with resistance training, this and previous studies may confirm that CA intensity adjusted to the individual's conditioning level may be an important factor for PAPE in physically active individuals.

Most studies observed PAPE for trained individuals (Wilson et al., 2013), as this phenomenon is related to the chronic adaptations in neuromuscular variables, arising from regular training which consequently favors greater resistance to fatigue, as well as predisposition to dissipate it more quickly (Boullosa et al., 2018). In this perspective, the significant improvement in CMJ performance for physically active individuals after running at 70% of MAS may be explained by submaximal intensity of the intervention. Moreover, prolonged exercises at submaximal intensities could delay the onset of fatigue, since slow-twitch muscle fibers are predominantly recruited during submaximal and prolonged events, and these fibers present greater resistance to fatigue (Boullosa et al., 2018; Hvid et al., 2013). On the other hand, previous studies have used brief and intense CAs (i.e., drop jumps) to improve repeated sprint ability (Zagatto et al., 2022), supramaximal cycling performance (De Poli et al., 2020) and 1000-m running performance (Boullosa et al., 2020b). Therefore, depending on the exercise mode, different stimuli may be effective to induce PAPE. Further studies are warranted to evaluate the best combination of running and plyometric exercises when looking for PAPE in athletes of different endurance sports.

In the present study, runners improved their CMJ performance after both running interventions (running at 70% of MAS and TTR). Although without significant differences, the highest enhancement was observed after the more intense exercise (TTR: ∆% = 6.94 vs. 70% of MAS: ∆% = 5.14). Evidence suggests that PAPE appearance can be more pronounced for endurance trained athletes (Boullosa et al., 2018), since trained individuals need a greater stimulus of CA to potentiate, while low submaximal intensities of CA can be insufficient to activate mechanisms responsible for PAPE (Zimmermann et al., 2020). For trained individuals, maximal exercises may present a greater stimulus in the activity of the neuromuscular system than submaximal exercises (Skof and Strojnik, 2007). Previously, Skof and Strojnik (2007) showed that well-trained runners performed a higher maximal contraction of quadriceps muscles after high-intensity running than after low-intensity running. Another possible mechanism for PAPE in trained individuals may be the improvement in elastic energy transfer in the CMJ (Vuorimaa et al., 2006). In this regard, Boullosa et al. (2011) observed enhancement in CMJ performance on a force plate after an incremental running test (UMTT) in endurance athletes and suggested that a possible explanation for potentiation was the counteracting force loss in the eccentric action with the subsequent increase in power production in the concentric action of the CMJ. As we used the flight time method for CMJ assessments, we cannot confirm the same mechanism in our data.

As the CMJ was performed after 2 min of the running interventions, the PAP mechanism (RLC) cannot be therefore discarded (Boullosa et al., 2020a). Enhancement in voluntary performance promoted by diverse metabolic, physiologic, neuromuscular and psychological factors, may occur at longer intervals (i.e., 5 to 12 min) (Seitz and Haff, 2016; Wilson et al., 2013), when PAP may already have importantly decreased (Zimmermann et al., 2020). Meanwhile, the influence of the PAP mechanism on the improvement in CMJ performance in participants evaluated cannot be confirmed. However, we did not perform the specific evaluation of the mechanism related to the PAP phenomenon (Prieske et al., 2020). While the difference between PAP and PAPE can objectively be described with the verification test (i.e., twitch verification vs. task performance), the associated mechanisms are not fully understood and can be the same in some cases (Boullosa et al., 2020a).

For SPRINTs, PAPE was not observed after the two running protocols for both groups, similarly to the study developed by Boullosa et al. (2011), in which endurance athletes at a group level maintained their performance in the SPRINT over 20 m after an incremental running test. However, other studies have observed a reduced SPRINT of 20 m after endurance running of 5 km (Nummela et al., 2008; Paavolainen et al., 1999). Importantly, SPRINT speeds (pre- and post-interventions) were faster for runners than for physically active participants, thus confirming an effect of training background on these performances.

No significant correlation was observed between general physical fitness (STEN score) and PAPE performances (∆%CMJ and ∆%SPRINT). In contrast, Vuorimaa et al. (2006) found that improvements in CMJ performances were positively correlated with the velocity associated to VO_2MAX_ (r = 0.44; *p*< 0.05) in middle- and long-distance runners. More recently, Guerra Junior et al. (2020) showed that combined body composition, muscle power and aerobic capacity showed a negative significant relationship with PAPE in soccer players. On the other hand, ∆%SPRINT performances were related to aerobic power and speed over 20 m in the current study. These results were expected since runners showed greater aerobic power than physically active individuals, as well as higher SPRINT speeds (pre- and post-interventions).

Interestingly, significant positive correlations were observed between training experience and ∆% CMJ in the TTR (r = 0.66; *p*< 0.05), and between training volume and ∆% CMJ in the TTR (r = 0.67; *p*< 0.05) for runners only. These results reinforce that athletes with more volume of endurance training could benefit more from PAPE after intense CAs. Previous studies have already shown that training status can influence the magnitude of PAPE (Chiu and Barnes, 2003; Wilson et al., 2013) in resistance trained individuals. Thus, this is the first study confirming that more trained runners present greater PAPE.

CMJ performance has been previously used to determine neuromuscular fatigue (Bosco et al., 1986), and it is an easy-to-perform test, since several methods are available such as contact mats and smartphone applications. Moreover, enhancement in elastic energy transfer may occur under a fatigued condition in the CMJ with both impairment (Bosco et al., 1986) and enhancement (Vuorimaa et al., 2006) in performance. For coaches, the CMJ performance evaluation can be a relevant tool to monitor runners’ training status as it reflects balance between muscular fatigue and potentiation.

The main limitation of the present study is related to the level of the runners assessed, since they were classified as recreational according to volume of training and the level of competitiveness (Mckinney et al., 2019). Future research with well-trained and elite runners is warranted to better understand the influence of training background and the performance level on these responses. Another limitation refers to the lack of physiological data (e.g., VO_2MAX_ and blood lactate concentrations) and PAP parameters to verify the contractile properties of the muscle.

## Conclusions

The present study demonstrated that both groups benefited from PAPE, after running CAs, when evaluated in the CMJ performance. For the SPRINT, both groups maintained speed in the test. The enhancement in CMJ performance was higher in the intervention with running at 70% of MAS for physically active individuals than in the TTR intervention. Meanwhile, for runners, both interventions improved the CMJ performance. Therefore, the results of this study suggest that the intensity of CA can influence PAPE according to the individual physical conditioning and the intensity of the protocols.
